# Supplementary material for: Fast ripples reflect increased excitability that primes epileptiform spikes
Source: medRxiv. 2023 Apr 19:2023.03.26.23287702. Originally published 2023 Mar 29. Preprint. [Version 2] doi: 10.1101/2023.03.26.23287702 (PMC10081394; doi:10.1101/2023.03.26.23287702)
Supplement: Supplement 1 [file media-1.pdf]

Supplementary Materials

Fast ripples reflect increased excitability that primes epileptiform spikes.

Shennan A Weiss[1,2,3], Itzhak Fried[5], Jerome Engel Jr[4,5,6,7,8], Michael R. Sperling[9], Robert K.S. Wong[2], Yuval Nir[10,11,12,13], Richard J Staba[4].

[1]Dept. of Neurology, [2]Dept. of Physiology and Pharmacology, State University of New York Downstate, Brooklyn, New York, 11203 USA
[3]Dept. of Neurology, New York City Health + Hospitals/Kings County, Brooklyn, NY, USA
[4]Dept. of Neurology, [5]Dept. of Neurosurgery, [6]Dept. of Neurobiology, [7]Dept. of Psychiatry and Biobehavioral Sciences, [8]Brain Research Institute, David Geffen School of Medicine at UCLA, Los Angeles, California, 90095, USA
[9]Depts. of Neurology and Neuroscience, Thomas Jefferson University, Philadelphia, Pennsylvania, 19107, USA
[10]Department of Physiology and Pharmacology, Sackler School of Medicine, Tel Aviv University, Tel Aviv 6997801, Israel
[11]Sagol School of Neuroscience, Tel Aviv University, Tel Aviv 6997801, Israel
[12]Department of Biomedical Engineering, Faculty of Engineering, Tel Aviv University, Tel Aviv 6997801, Israel
[13]The Sieratzki-Sagol Center for Sleep Medicine, Tel Aviv Sourasky Medical Center, Tel Aviv 6423906, Israel

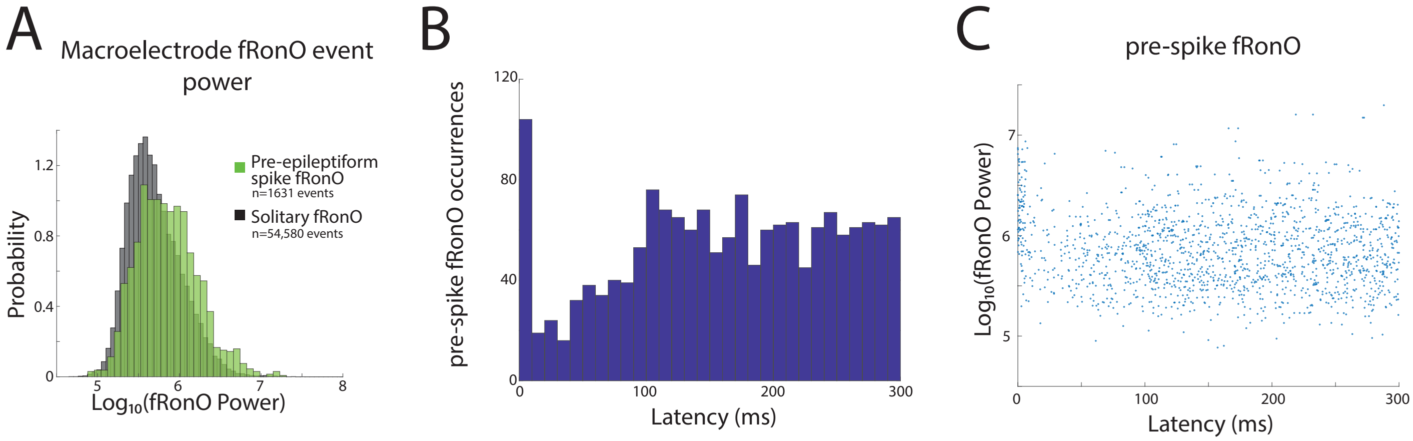

Figure S1: An examination pre-epileptiform spike fast ripple on oscillation (fRonO) power with increased temporal resolution. (A) Normalized histogram of fRonO event spectral power. fRonO events preceding spikes had a larger power (t-test, p<1e-10, Cohen's d=.475) than solitary fRonO. (B) Histogram of the latency in milliseconds (ms) between pre-spike fRonO and the after-going spike. Note that a relatively small, but distinct, subpopulation of events occurred within <10 msec of the spike. (C) Plot of the pre-spike fRonO power as a function of latency. Note that relatively larger powers (see A) were seen across a broad range of latencies. However, fRonO events occurring <10 ms before the after-going spike had the largest power distribution

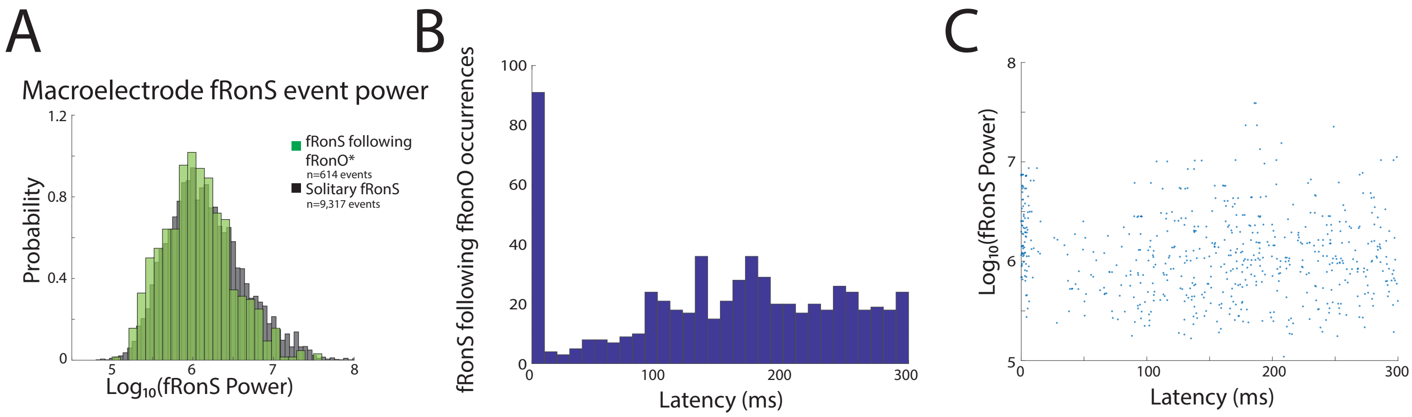

Figure S2: An examination of fast ripple on spike (fRonS) following fast ripple on oscillation (fRonO) power with increased temporal resolution. (A) Normalized histogram of fRonS event power in macroelectrode recordings. fRonS power was reduced when it followed a fRonO (t-test, p<1e-5, Cohen's d=0.21) (B) Histogram of the latency in milliseconds (ms) between the fRonO and the after-going fRonS. Note that a distinct subpopulation of events occurred within <10 msec of the spike. (C) Plot of the fRonS following fRonO power as a function of latency. Note that relatively decreased powers (see A) were seen across a broad range of latencies. However, fRonS events following a fRonO by <10 ms had the largest power distribution suggesting a distinct mechanism.

Table S1: Patient characteristics in the resection Table S4: Results of generalized linear mixed effects model for the outcome of fast ripple (FR) power (arbitrary units A.U.) with random effect term of electrode contact (i.e., node) pair (i.e., edge) number. The fixed effects of the model were 1) whether the fast ripple was recorded from the out-node or in-node (abbr., inout); 2) whether the fast ripple was considered propagating from the sign test either from the out-node (abbr., prop); 3) the Euclidian distance of propagation between the in- and out nodes; 4) whether the out- or in-node was located in the seizure-onset zone (abbr., soz); d.f.=39,001. Brackets indicate

ID	Risk Factor	MRI	PET (hypo-metabolic)	iEEG clinical consensus SOZs	Surgery	Path.	Outcome
1.	minor TBI	Normal	L tempo-ral	L MT	modified L ATL hippocampus sparing	Gliosis	Engel IA@24 months
2.	hyperten-sive enceph-alopathy	post L ATL	N/A	L middle tem-poral gyrus	modified L temporal lo-bectomy (posterior tem-poral included)	Gliosis	Engel 1A @48 months
3.	Minor TBI	Normal	Normal	Right insula, cuneus, inferi-or and middle frontal gyrus	R. Frontal lobe	Gliosis	Engel IA@24 months
4.	None	Normal	R temporal	R Inferior tem-poral gyrus	modified R temporal lo-bectomy (posterior tem-poral included)	Gliosis	Engel IA@24 months
5.	None	L MTL whitematter hyper-inten-sity	Normal	R SMA	R frontal lobe resection	cortical dyspla-sia	Engel IA@24 months
6.	None	Normal	Normal	L cingulate gyrus, medial frontal gyrus, middle frontal gyrus, superior frontal gyrus	L frontal lobec-tomy	cortical dyspla-sia	Engel IA@40 months
7.	meningitis	Encepha-lo-malacia	L temporal	L MT, uncus, superior tem-poral gyrus, frontal lesion	L temporal and frontal lobe resection	gliosis	Engel IB@42 months
8.	none	1 cm pineal cyst	R lateral temporal	L MT	Modified L temporal lo-bectomy (pos-terior temporal included)	Gliosis	Engel IIB@24 months

9.	None	T2 hyper-intensity in R temporal pole > L frontal pole. Inferior portion of R temporal pole with blurred gray-white matter border	R temporal	R MT	R anterior ATL	Cortical dysplasia IIb	Engel IA@60 months
10.	None	Normal	R temporal	Bilateral MT, middle temporal gyrus R>L	modified R ATL (preserved middle and superior temporal gyrus)	gliosis	Engel IVC@48 months
11.	TBI, family history	left superior temporal gyrus encephalomalicia	L parieto-occipital	L temporal neocortical, L frontal	modified LATL hippocampus sparing	gliosis	Engel IV@6 months RNS placed and revised
12.	None	Normal	L temporal	R fusiform gyrus, superior temporal gyrus, uncus	R ATL	MTS	Engel IB@35 months
13.	TBI w/ LOC	L MTS, extra-temporal T2	L temporal and frontal	L MT, fusiform gyrus, uncus	L MT visualase	N/A	Engel IIIA@18 months
14.	None	periventricular nodular heterotopia, right frontal T2	R temporal	R MT	ATL	gliosis	Engel IB@31 months
15.	TBI w/ LOC	Encephalomalacia	R temporal	R insula, bilateral middle temporal gyrus, superior temporal gyrus	modified R ATL (posterior temporal and temporal-parietal-occipital junction included.	gliosis	Engel IVB@33 months

16.	None	Prior R. ATL	N/A	Right orbitofrontal cortex.	R. Frontal lobe	hippocampal sclerosis, cortical dysplasia	Engel IVB@24 months
17.	encephalitis	Encephalomalacia	Normal	L inferior frontal gyrus, insula, MT	L temporal lobe and insula resection	Gliosis	SUDEP @6 weeks
18.	Significant head injury with LOC	Left temporal T2 hyperintensity with mild enhancement	N/A	Bilateral MT, right lateral temporal	L temporal lobectomy, anterior thalamic DBS	Gliosis	Engel IVB@24 months
19.	None	R parietal lobe resection	R parietal and R occipital	R insula, precuneus, middle occipital gyrus, superior parietal lobule, superior occipital gyrus, superior temporal gyrus, middle temporal gyrus	R parietal	gliosis	Engel IIIA@18 months
20.	None	L posterior fossa arachnoid cyst, R ATL	R temporal	L MT, R cingulate, post. cingulate, mesial frontal, precuneus	R anterior cingulate thermal ablation	gliosis	Engel IVB@36 months
21.	None	Prior R parietal resection	R parietal and occipital hypometabolism	R parietal lobe	R. Parietal lobe resection	Gliosis	Engel IVB@36 months
22	TBI	left MTS	normal	L MT	L ATL	gliosis hippocampal sclerosis	Engel IIIA@63 months
23	febrile seizures	prior hippocampal sparing temporal lobectomy	N/A	R anterior cingulate, MT, uncus	R ATL	gliosis	Engel IVB@40 months

Table S2: Results of generalized linear mixed effects model for the outcome of the maximum unit firing rate during the fast ripple on oscillation (fRonO) detected in the macroelectrode, (action potentials/sec) after Gaussian smoothing, minus the mean baseline unit firing rate measured 750 msec before the fRonO for each peri-fRonO trial (d.f.=224,266). The random effect terms were patient id, macroelectrode contact ID of the Behnke-Fried electrode, and the unit id to determine that the effects were observed across all units. The fixed effects of the model were 1) the log10(power) of the trial's fRonO measured in arbitrary units, and 2) whether, in the trial, the fRonO preceded a sharply contoured epileptiform spike, with or without a high-frequency oscilation, within 300 msec. ":" refers to interaction term. Abbreviations CI: confidence interval.

fRonO unit firing rate - baseline firing rate Fixed effect name	Estimate [95% CI]	tStat	p-value
(intercept)	-8.528 [-9.012 -8.043]	-34.472	<1e-100
1. power	1.044 [0.997 1.089]	46.026	<1e-100
2. preSpike	1.740 [0.764 2.716]	3.493	<1e-3
3. preSpike:power	-0.233 [-0.392 -0.734]	-2.863	<1e-2

Table S3: Results of generalized linear mixed effects model for the outcome of the mean baseline unit firing rate measured 750 msec before the fast ripple on oscillation (fRonO) for each peri-fRonO trial (d.f.=224,266). The random effect terms were patient id, macroelectrode contact ID of the Behnke-Fried electrode, and the unit id to determine that the effects were observed across all units. The fixed effects of the model were 1) the log10(power) of the trial's fRonO measured in arbitrary units, and 2) whether, in the trial, the fRonO preceded a sharply contoured epileptiform spike, with or without a high-frequency oscilation, within 300 msec. ":" refers to interaction term. Abbreviations CI: confidence interval.

pre-fRonO<br>baseline unit firing rate	Estimate [95% CI]	tStat	p-value
(intercept)	-1.326 [-1.680 -0.972]	-7.353	<1e-12
1. power	-0.030 [-0.040 -0.019]	-5.426	<1e-7
2. preSpike	0.145 [-0.086 0.381]	1.240	n.s.
3. preSpike:power	-0.023 [-0.063 0.017]	-1.141	n.s.

Table S4: Results of generalized linear mixed effects model for the outcome of the maximum unit firing rate during the ripple on oscillation (RonO) detected in the macroelectrode, (action potentials/sec) after Gaussian smoothing, minus the mean baseline unit firing rate measured 750 msec before the RonO for each peri-RonO trial (d.f.=2,603,844). The random effect terms were patient id, macroelectrode contact ID of the Behnke-Fried electrode, and the unit id to determine that the effects were observed across all units. The fixed effects of the model were 1) the log10(power) of the trial's RonO measured in arbitrary units, and 2) whether, in the trial, the RonO preceded a sharply contoured epileptiform spike, with or without a high-frequency oscilation, within 300 msec. ":" refers to interaction term. Abbreviations CI: confidence interval.

RonO unit firing rate - baseline firing rate<br>Fixed effect name	Estimate [95% CI]	tStat	p-value
(intercept)	-7.909 [-8.367 -7.450]	-33.823	<1e-100
1. power	0.800 [0.788 0.811]	131.56	<1e-100
2. preSpike	-0.514 [-0.822 -0.206]	-3.270	<5e-3
3. preSpike:power	0.095 [0.0501 0.139]	4.170	<1e-4

Table S5: Results of generalized linear mixed effects model for the outcome of the mean baseline unit firing rate measured 750 msec before the ripple on oscillation (RonO) for each peri-RonO trial (d.f.=2,603,845). The random effect terms were patient id, macroelectrode contact ID of the Behnke-Fried electrode, and the unit id to determine that the effects were observed across all units. The fixed effects of the model were 1) the log10(power) of the trial's RonO measured in arbitrary units, and 2) whether, in the trial, the RonO preceded a sharply contoured epileptiform spike, with or without a high-frequency oscilation, within 300 msec. ":" refers to interaction term. Abbreviations CI: confidence interval.

pre-RonO<br>baseline unit firing rate	Estimate [95% CI]	tStat	p-value
(intercept)	-1.858 [-2.123 -1.592]	-13.729	<1e-42
1. power	-0.020 [-0.024 -0.017]	-10.495	<1e-25
2. preSpike	-0.096 [-0.177 -0.014]	-2.296	<0.05
3. preSpike:power	0.016 [0.004 0.028]	2.568	<0.05

Table S6: Results of generalized linear mixed effects model for the outcome of the maximum unit firing rate during the fast ripple on spike (fRonS) detected in the macroelectrode, (action potentials/sec) after Gaussian smoothing, minus the mean baseline unit firing rate measured 750 msec before the fRonS for each peri-fRonS trial (d.f.=48,164). The random effect terms were patient id, macroelectrode contact ID of the Behnke-Fried electrode, and the unit id to determine that the effects were observed across all units. The fixed effects of the model were 1) the log10(power) of the trial's fRonS measured in arbitrary units, and 2) whether, in the trial, the fRonS followed a fast ripple on oscillation (fRonO) within 300 msec. ":" refers to interaction term. Abbreviations CI: confidence interval.

fRonS unit firing rate - baseline firing rate<br>Fixed effect name	Estimate [95% CI]	tStat	p-value
(intercept)	-1.887 [-2.42 -1.34]	-6.846	<1e-11
1. power	0.125 [0.105 0.144]	12.464	<1e-34
2. followingFRonO	-2.830 [-3.662 -2.0]	-6.657	<1e-10
3. followingFRonO:power	0.430 [0.301 0.559]	6.549	<1e-10

Table S7: Results of generalized linear mixed effects model for the outcome of the maximum unit firing rate during the fast ripple on spike (fRonS) detected in the macroelectrode, (action potentials/sec) after Gaussian smoothing, minus the mean baseline unit firing rate measured 750 msec before the fRonS for each peri-fRonS trial (d.f.=48,164). The random effect terms were patient id, macroelectrode contact ID of the Behnke-Fried electrode, and the unit id to determine that the effects were observed across all units. The fixed effects of the model were 1) the log10(power) of the trial's fRonS measured in arbitrary units, and 2) whether, in the trial, the fRonS followed a ripple on oscillation (RonO) within 300 msec. ":" refers to interaction term. Abbreviations CI: confidence interval.

fRonS unit firing rate - baseline firing rate Fixed effect name	Estimate [95% CI]	tStat	p-value
(intercept)	-2.019 [-2.565 -1.473]	-7.250	<1e-12
1. power	0.141 [0.120 0.162]	13.14	<1e-38
2. followingRonO	0.307 [0.048 0.565]	2.34	<0.05
3. followingRonO:power	-0.036 [-0.077 0.005]	1.73	n.s.

Table S8: Results of generalized linear mixed effects model for the outcome of the mean baseline unit firing rate measured 750 msec before the fast ripple on spike (fRonS) for each peri-fRonS trial (d.f.=48,164). The random effect terms were patient id, macroelectrode contact ID of the Behnke-Fried electrode, and the unit id to determine that the effects were observed across all units. The fixed effects of the model were 1) the log10(power) of the trial's fRonS measured in arbitrary units, and 2) whether, in the trial, the fRonS followed a fast ripple on oscillation (fRonO) within 300 msec. Abbreviations CI: confidence interval.

pre-fRonS<br>baseline unit firing rate	Estimate [95% CI]	tStat	p-value
(intercept)	-1.408 [1.902 -0.914]	-5.589	<1e-7
1. power	-0.0123 [-0.024 -0.001]	-2.105	<0.05
2. followingFRonO	-0.742 [-0.896 -0.049]	-2.186	<0.05
3. followingFRonO:power	0.0720 [0.004 0.14]	2.062	<0.05

Table S9: Results of generalized linear mixed effects model for the outcome of the mean baseline unit firing rate measured 750 msec before the fast ripple on spike (fRonS) for each peri-fRonS trial (d.f.=48,164). The random effect terms were patient id, macroelectrode contact ID of the Behnke-Fried electrode, and the unit id to determine that the effects were observed across all units. The fixed effects of the model were 1) the log10(power) of the trial's fRonS measured in arbitrary units, and 2) whether, in the trial, the fRonS followed a ripple on oscillation (RonO) within 300 msec. Abbreviations CI: confidence interval.

pre-fRonS<br>baseline unit firing rate	Estimate [95% CI]	tStat	p-value
(intercept)	-1.392 [-1.887 -0.900]	-5.519	<1e-7
1. power	-0.017 [-0.030 -0.003]	-2.452	<0.05
2. followingRonO	-0.103 [-0.247 0.040]	-1.408	n.s.
3. followingRonO:power	0.022 [-0.0004 0.044]	1.918	n.s.

Table S10: Results of generalized linear mixed effects model for the outcome of the maximum unit firing rate during the ripple on spike (RonS) detected in the macroelectrode, (action potentials/sec) after Gaussian smoothing, minus the mean baseline unit firing rate measured 750 msec before the RonS for each peri-RonS trial (d.f.=391,663). The random effect terms were patient id, macroelectrode contact ID of the Behnke-Fried electrode, and the unit id to determine that the effects were observed across all units. The fixed effects of the model were 1) the log10(power) of the trial's RonS measured in arbitrary units, and 2) whether, in the trial, the RonS followed a fast ripple on oscillation (fRonO) within 300 msec. ":" refers to interaction term. Abbreviations CI: confidence interval.

RonS unit firing rate -<br>baseline firing rate<br>Fixed effect name	Estimate [95% CI]	tStat	p-value
(intercept)	-3.272 [-3.693 -2.852]	-15.253	<1e-51
1. power	0.260 [0.250 0.270]	50.411	<1e-100
2. followingFRonO	-2.684 [-3.225 -2.144]	-9.738	<1e-21
3. followingFRonO:power	0.372 [0.298 0.445]	9.862	<1e-22

Table S11: Results of generalized linear mixed effects model for the outcome of the maximum unit firing rate during the ripple on spike (RonS) detected in the macroelectrode, (action potentials/sec) after Gaussian smoothing, minus the mean baseline unit firing rate measured 750 msec before the RonS for each peri-RonS trial (d.f.=391,663). The random effect terms were patient id, macroelectrode contact ID of the Behnke-Fried electrode, and the unit id to determine that the effects were observed across all units. The fixed effects of the model were 1) the log10(power) of the trial's RonS measured in arbitrary units, and 2) whether, in the trial, the RonS followed a ripple on oscillation (RonO) within 300 msec. ":" refers to interaction term. Abbreviations CI: confidence interval.

RonS unit firing rate - baseline firing rate Fixed effect name	Estimate [95% CI]	tStat	p-value
(intercept)	-3.183 [-3.603 -2.763]	-14.861	<1e-49
1. power	0.245 [0.236 0.259]	42.905	<1e-100
2. followingRonO	-0.535 [-0.691 -0.380]	-6.733	<1e-10
3. followingRonO:power	0.076 [0.055 0.098]	6.923	<1e-11

Table S12: Results of generalized linear mixed effects model for the outcome of the mean baseline unit firing rate measured 750 msec before the fast ripple on spike (RonS) for each peri-RonS trial (d.f.=391,663). The random effect terms were patient id, macroelectrode contact ID of the Behnke-Fried electrode, and the unit id to determine that the effects were observed across all units. The fixed effects of the model were 1) the log10(power) of the trial's RonS measured in arbitrary units, and 2) whether, in the trial, the RonS followed a fast ripple on oscillation (fRonO) within 300 msec. Abbreviations CI: confidence interval.

pre-RonS<br>baseline unit firing rate	Estimate [95% CI]	tStat	p-value
(intercept)	-1.510 [-1.935 -1.084]	-6.956	<1e-11
1. power	-0.026 [-0.031 -0.022]	-11.119	<1e-27
2. followingFRonO	-0.671 [-0.846 -0.495]	-7.499	<1e-13
3. followingFRonO:power	0.099 [0.743 0.124]	7.825	<1e-14

Table S13: Results of generalized linear mixed effects model for the outcome of the mean baseline unit firing rate measured 750 msec before the fast ripple on spike (RonS) for each peri-RonS trial (d.f.=391,663). The random effect terms were patient id, macroelectrode contact ID of the Behnke-Fried electrode, and the unit id to determine that the effects were observed across all units. The fixed effects of the model were 1) the log10(power) of the trial's RonS measured in arbitrary units, and 2) whether, in the trial, the RonS followed a ripple on oscillation (RonO) within 300 msec. Abbreviations CI: confidence interval.

pre-RonS<br>baseline unit firing rate	Estimate [95% CI]	tStat	p-value
(intercept)	-1.544 [-1.968 -1.120]	-7.135	<1e-12
1. power	-0.023 [-0.029 -0.018]	-8.499	<1e-16
2. followingRonO	-0.014 [-0.080 0.053]	-0.402	n.s.
3. followingRonO:power	0.010 [0 0.020]	2	n.s.
